# Loss‐of‐Function Mutations in the *ALPL* Gene Presenting with Adult Onset Osteoporosis and Low Serum Concentrations of Total Alkaline Phosphatase

**DOI:** 10.1002/jbmr.3928

**Published:** 2020-01-07

**Authors:** Nerea Alonso, Beatriz Larraz‐Prieto, Kathryn Berg, Zoe Lambert, Paul Redmond, Sarah E Harris, Ian J Deary, Carys Pugh, James Prendergast, Stuart H Ralston

**Affiliations:** ^1^ Rheumatology and Bone Disease Unit, Centre for Genomic and Experimental Medicine, MRC institute of Genetics and Molecular Medicine University of Edinburgh Edinburgh UK; ^2^ Department of Psychology University of Edinburgh Edinburgh UK; ^3^ Centre for Cognitive Ageing and Cognitive Epidemiology University of Edinburgh Edinburgh UK; ^4^ Division of Psychiatry University of Edinburgh, Royal Edinburgh Hospital Edinburgh UK; ^5^ Genetics and Genomics Division The Roslin Institute, University of Edinburgh Midlothian UK

**Keywords:** ALKALINE PHOSPHATASE, *ALPL* VARIANTS, BISPHOSPHONATES, HYPOPHOSPHATASIA, OSTEOPOROSIS

## Abstract

Hypophosphatasia (HPP) is a rare inherited disorder characterized by rickets and low circulating concentrations of total alkaline phosphatase (ALP) caused by mutations in *ALPL*. Severe HPP presents in childhood but milder forms can present in adulthood. The prevalence and clinical features of adult HPP are poorly defined. The aim of this study was to evaluate the prevalence and clinical significance of low serum total alkaline phosphatase (ALP) levels in a clinic‐based population of adult osteoporotic patients. We searched for patients with low ALP in a cohort of 3285 patients referred to an osteoporosis clinic over a 10‐year period and performed mutation screening of *ALPL* in those with low ALP (≤40 U/L) on two or more occasions. These individuals were matched with four clinic controls with a normal ALP. We also evaluated the prevalence of low ALP and *ALPL* mutations in 639 individuals from the general population from the same region. We identified 16/3285 (0.49%) clinic patients with low ALP and 14 (87.5%) had potentially pathogenic variants in *ALPL*. Eight of these individuals were heterozygous for mutations previously described in HPP and 2 were heterozygous for novel mutations (p.Arg301Trp and p.Tyr101X). These mutations were not found in clinic controls or in the general population. Eight patients with low ALP, including 4 with *ALPL* mutations, were treated with bisphosphonates for an average of 6.5 years. In these individuals, the rate of fractures during treatment was comparable to that in normal ALP clinic controls who were treated with bisphosphonates. We conclude that heterozygous loss‐of‐function mutations in *ALPL* are common in osteoporosis patients with low ALP. Further studies are required to determine how best these individuals should be treated. © 2019 The Authors. *Journal of Bone and Mineral Research* published by American Society for Bone and Mineral Research.

## Introduction

Hypophosphatasia (HPP) is a rare inherited disorder characterized by low circulating concentrations of total alkaline phosphatase (ALP), rickets, muscular hypotonia, seizures, and multiple fractures.^1^ The severity of the disease ranges widely from a severe, recessively inherited disorder presenting neonatally, which until recently was fatal without treatment, to milder forms that can present later in childhood or in adults. In Europe, severe HPP has been estimated to affect about 1 in 300,000 individuals.^2^

Adults with HPP may present with osteomalacia, osteoporosis, fragility fractures, and dental problems. Atypical femoral fractures have been reported in some patients who have been treated with bisphosphonates.^3^, ^4^, ^5^, ^6^ The prevalence and mode of clinical presentation in adults with HPP is unclear. It has been suggested to affect about 1 in 6000 individuals^2^ but may be more common due to the diagnosis being overlooked.^7^

The cause of HPP is loss‐of‐function or dominant negative mutations in *ALPL*.^8^ Based on the population prevalence of recessive HPP, the proportion of dominant mutations, and the penetrance of the *ALPL* mutations,^2^ one would expect about 1 in 500 individuals in the general population to be carriers, although it is unclear to what extent these individuals are symptomatic.^9^ The *ALPL* gene encodes the tissue‐nonspecific ALP, a 50 kDa protein expressed in bone, liver, and kidney. In bone, it is essential for the mineralization process, because it hydrolyzes pyrophosphate, an inhibitor of hydroxyapatite formation and, at the same time, releases inorganic phosphate, which combines with calcium to promote mineralization.^10^ To date, at least 397 variants have been described in this gene as causing HPP (as reported in The Tissue Nonspecific Alkaline Phosphatase Gene Mutation Database).^11^ Most are missense mutations found in homozygous or compound heterozygous form. Additionally, low‐frequency, heterozygous missense variants in *ALPL* have been observed in men with low bone mineral density^12^ and at low frequency in adults with various rheumatologic disorders, including osteoarthritis, rheumatoid arthritis, Castleman disease, and patients with recurrent fractures.^13^

The prevalence of low ALP levels and its clinical significance in osteoporotic patients have not yet been studied. Therefore, the aim of this study was to investigate how frequently low ALP occurs in patients with osteoporosis and to determine the proportion of these individuals who have mutations in *ALPL*.

## Materials and Methods

The study was based at the osteoporosis clinic at the Western General Hospital in Edinburgh (UK), which is a secondary care referral center for patients with bone disease. In this clinic, total ALP concentrations are measured in all patients as part of a routine biochemical screen in the NHS Lothian laboratories. In keeping with the suggestion by Lee and colleagues,^14^ we derived an assay‐specific reference range for ALP by analysis of samples from 200 healthy controls from the local population. We searched the clinic database for individuals who had ALP values below the lower limit of the reference range (≤40 U/L) on two or more occasions. We also screened a population‐based cohort from the same region—the Lothian Birth Cohort of 1936 (LBC1936)^15^ —for low ALP levels that had been measured on two occasions by the same laboratory as part of a routine biochemical screen (*n* = 639). In both cohorts, we screened for coding variants of *ALPL* according to standard techniques. Full details of the genotyping methodologies used are provided in the Supplemental Materials and Methods.

Comparison between groups for categorical variables was by chi‐square test or Fisher's exact test and by nonparametric Mann–Whitney *U* test for continuous variables, with the exception of fractures per patient per year, which was calculated using the Kruskal‐Wallis test. The *p* value for significance was set at 0.05. Statistical analysis of the data was performed using SPSS version 21 (IBM Corp., Armonk, NY, USA).

## Results

### Clinical characteristics of low ALP cases and clinic controls

A search of the osteoporosis clinic database identified 16/3285 (0.49%) individuals with low ALP values (≤40 U/L) on at least two occasions. The clinical characteristics of patients with low ALP levels were similar to those of clinic controls in terms of bone mineral density (BMD), fracture history, smoking, alcohol intake, and comorbidity (Table 1). Eight individuals with low ALP had been treated with bisphosphonates before being referred to the clinic (6 with alendronic acid and 2 with risedronate), but serum ALP levels were low in 7 of these individuals before starting bisphosphonates. In 1 patient, ALP had not been checked before starting risedronate (Table 1). The mean ± SD ALP values before starting bisphosphonate therapy in the 7 individuals where data were available was 34 ± 6.07 U/L compared with 29 ± 4.01 U/L at the time of clinic referral, a difference that was not significant (*p* = 0.11). None of the individuals with low ALP had clinical features to suggest hypophosphatasia, such as metatarsal fractures, atypical femoral fracture, chondrocalcinosis, premature tooth loss, or a family history of HPP.

**Table 1:** Clinical Characteristics of Low ALP Clinic Cases and Controls

Clinical characteristics	Low ALP cases (*n* = 16)	Normal ALP controls (*n* = 64)
Age (years)	66 ± 3.0	64 ± 1.3
Body mass index (kg/m^2^)	23.4 ± 1.2	24.9 ± 0.7
ALP (U/L)	29.7 ± 0.82	88.0 ± 1.7*
Previous bisphosphonate therapy, *n* (%)	8 (50)	18 (28.1)
Female sex, *n* (%)	13 (81.2)	52 (81.2)
Age at menopause (years)	49 ± 1.6	46 ± 1.0
Serum 25‐vit D (nmol/L)	54.7 ± 11.8	51.3 ± 5.5
Serum albumin (g/dL)	4.04 ± 0.07	4.29 ± 0.05**
Estimated glomerular filtration rate (mL/min)	56.5 ± 2.5	59.4 ± 0.6
Alcohol intake (units)	5.7 ± 1.8	6.2 ± 1.2
Smoker, *n* (%)	1 (6.2)	18 (28.1)
Lumbar spine bone mineral density (BMD)	0.731 ± 0.03	0.797 ± 0.02
Femoral neck BMD	0.589 ± 0.02	0.621 ± 0.03
Total hip BMD	0.716 ± 0.02	0.686 ± 0.03
FRAX major fracture	16.1 ± 2.10	14.2 ± 1.14
FRAX hip fracture	6.3 ± 1.64	4.9 ± 0.67
Age at first fracture (years)	62.3 ± 3.8	61.1 ± 2.2
Nonvertebral fractures,^a^ *n* (%)	8 (50.0)	24 (37.5)
Vertebral fracture, *n* (%)	8 (50.0)	23 (36.0)
Early menopause, *n* (%)	3 (18.7)	8 (12.5)
Charlson index	3.06 ± 0.64	3.11 ± 0.24

a Low‐trauma fractures validated by X‐ray.

* *p* = 7.30 × 10^−10^.

** *p* = 0.009.

By way of comparison, 18/64 (28.1%) of controls had previously received bisphosphonate, a proportion that was not significantly different from low ALP cases (*p* = 0.10). Serum albumin concentrations were marginally lower in those with low ALP (4.04 ± 0.07 versus 4.29 ± 0.05 g/L, *p* = 0.009), but no differences were observed in other routine biochemistry (not shown). One clinic control with normal ALP values had presented an atypical femoral fracture associated with bisphosphonate treatment.

### Genetic analysis of low ALP cases and clinic controls

**Figure 1:**
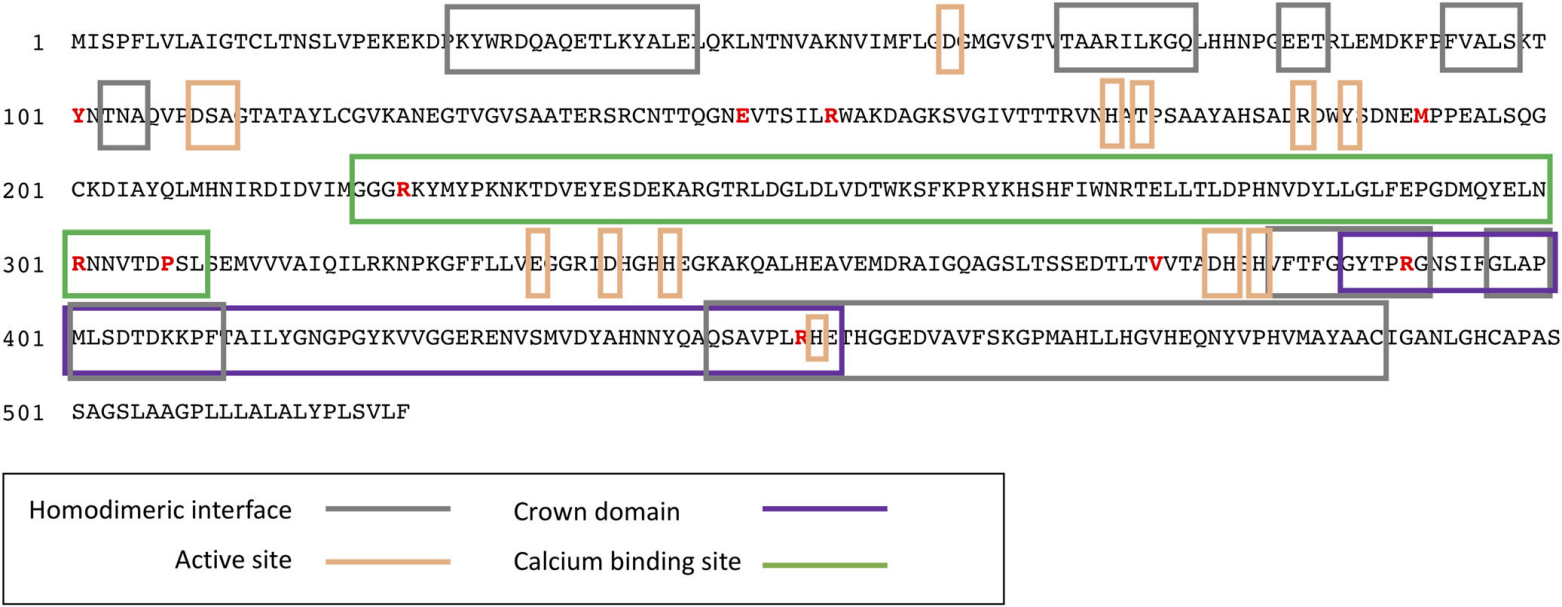
Location of the *ALPL* variants found in this study. The location of pathogenic missense variants found in osteoporotic patients with low ALP levels are highlighted in red. The protein sequence and domains have been adapted from Silvent and colleagues.^22^

We found that 14/16 (87.5%) individuals with low ALP had potentially pathogenic variants in *ALPL* (Table 2 and Supplemental Table S3). Eight patients had *ALPL* mutations that have already been reported in HPP and 2 further patients had novel variants predicted to be pathogenic (c.303c > a, causing a nonsense change p.Tyr101X, and c.901a > t, encoding a missense mutation p.Arg301Trp). All variants were found in heterozygous form. The frequency of *ALPL* mutations in the low ALP clinic cases was significantly higher than in the clinical controls with normal ALP (*p* = 4.86 × 10^−9^). Four low ALP clinic patients carried the p.Arg152His polymorphism compared with 1 normal ALP clinic control (1.5%), a difference that was significant (*p* = 0.005). The variants were located at different sites in the ALP protein, many affecting regions of functional importance (Fig. 1). *In silico* analysis of the missense p.Arg301Trp variant predicted that the change was pathogenic (Supplemental Table S2). The variant affects amino acid 301, which is within a Ca^++^ binding site of the protein (Fig. 1) and which is highly conserved through evolution (Supplemental Fig. S1). In addition to these functional variants, we found a common benign variant (p.Val522Ala, rs34605986) in 2/16 (12.5%) low ALP patients and 17 clinic controls (26.5%), a difference that was not significant.

**Table 2:** Variants Found in *ALPL* Gene in Clinic Patients With Low ALP

ID	ALP^a^ (U/L)	*ALPL* variant	Exon	Protein change	Comment
1	32	c.575 t > c	6	p.Met192Thr	Infantile HPP; compound heterozygote (SESEP‐University of Versailles‐Saint Quentin)
2	27.5	c.303c > a	4	p.Tyr101X	Novel
3	32.5	c.668 g > a	7	p.Arg223Gln	Perinatal HPP; found with a large deletion^16^, ^17^
4	29.5	c.455 g > a	5	p.Arg152His	Polymorphism; affecting a residue involved in odonto‐HPP^18^ low BMD^12^
5	34	c.920c > t	9	p.Pro307Leu	Infantile HPP; compound heterozygote (SESEP‐University of Versailles‐Saint Quentin)
6	26	c.1171c > t	10	p.Arg391Cys	Childhood HPP; compound heterozygote^19^, ^20^
7	34.5	c.455 g > a	5	p.Arg152His	Polymorphism; affecting a residue involved in odonto‐HPP;^18^ low BMD^12^
8	21.5	c.436 g > a	5	p.Glu146Lys	Asymptomatic; low BMD^12^
9	29.5	‐	‐	‐	
10	33	c.436 g > a	5	p.Glu146Lys	Asymptomatic; low BMD^12^
11	28.5	c.901a > t	9	p.Arg301Trp	Novel
12	30	‐	‐	‐	
13	32.5	c.455 g > a	5	p.Arg152His	Polymorphism; affecting a residue involved in odonto‐HPP;^18^ low BMD^12^
14	26	c.1120 g > a	10	p.Val374Met	Adult HPP; compound heterozygote (SESEP‐University of Versailles‐Saint Quentin^17^)
15	28.5	c.1348c > t	12	p.Arg450Cys	Infantile HPP; homozygous^20^, ^21^
16	30	c.455 g > a	5	p.Arg152His	Polymorphism; affecting a residue involved in odonto‐HPP;^18^ low BMD^12^

a Mean of two independent ALP readings.

### Frequency of low ALP and *ALPL* variants in the general population

We identified 6/639 individuals (0.9%) with low ALP (≤40 U/L) in the LBC1936 cohort. None of the individuals in LBC1936 were known to have a diagnosis of HPP and none had reported premature tooth loss. Information on AFF, metatarsal fractures, and chondrocalcinosis was not available. Genetic analysis identified the p.Arg152His polymorphism (rs149344982) in heterozygous form in 31/639 (4.8%) participants in LBC1936. Individuals carrying the “A” variant (encoding Histidine) had significantly lower levels of ALP than those with the “G” variant, encoding Arginine (mean ± SEM ALP = 60.4 ± 3.2 versus 79.0 ± 1.2 U/L; *p* = 3.58 × 10^−4^). Two of the 31 individuals with the “A” variant showed low ALP levels (≤40 U/L). No individuals in the 1936 LBC cohort were found to have *ALPL* mutations.

The p.Val522Ala polymorphism (rs34605986) was found in 18.5% of LBC1936, in both homozygous and heterozygous forms. No significant differences in ALP levels were found between individuals of different genotype (ALP = 77.7 ± 1.30 for the TT genotype; 79.3 ± 2.01 for the CT genotype, 81.1 ± 6.4 for the CC genotype, *p* value = 0.836).

### Treatment and clinical outcome in low ALP cases and controls

Details of the treatments given to the low ALP cases and controls are shown in Table 3. Bisphosphonates were the most commonly prescribed treatment in both groups. During follow‐up of up to 9 years, there was no significant difference between the patients with low ALP and controls in the proportion of patients who had suffered one or more fractures. One atypical femoral fracture was observed in the control group.

**Table 3:** Treatment and Clinical Outcome in Low ALP Cases and Clinic Controls

Treatments	Cases (*n* = 16)	Controls (*n* = 64)	*p* Value
Alendronic acid	8 (50.0%)	37 (56.1%)	0.22
Risedronate	0 (0%)	5 (7.6%)	
Zoledronic acid	2 (14.2%)	10 (15.1%)	
Denosumab	2 (14.2%))	3 (4.5%)	
Teriparatide	1 (7.6%)	0 (0%)	
Hormone replacement therapy	0 (0%)	1 (1.5%)	
No treatment	3 (21.8%)	10 (15.1%)	
Duration of follow‐up (years)	6.5 ± 3.4	9.2 ± 2.6	0.001
Fractures during follow‐up			0.28
No fractures	10 (71.4%)	33 (50.0%)	
1 fracture	3 (21.4%)	18 (27.3%)	
2 fractures	1 (7.1%)	11 (16.7%)	
3 fractures	0 (0%)	4 (6.1%)	
Fracture/patient/year	0 (0–0.07)	0.04 (0–0.17)	0.09
Atypical femoral fractures	0 (0%)	1 (1.6%)	1.0

Values are mean ± SD or *n* (%), except fractures/patient/year, which shows median and interquartile ranges. The *p* values refer to differences between groups calculated by chi‐square test or Fisher's exact test (fracture and treatments), *t* test (duration of follow‐up), and fractures per patient per year (Kruskal–Wallis test).

## Discussion

We have found that about 0.5% of patients attending our osteoporosis clinic had low circulating concentrations of ALP and that the majority of these individuals had pathogenic mutations in *ALPL*. Although 8 patients had previously been treated with bisphosphonates, 7 of these individuals had ALP measured before starting treatment and it was low in all cases. In 8/16 clinic cases with low ALP, known loss‐of‐function mutations in *ALPL* were found and, in a further 2 cases, novel variants were found and predicted to be pathogenic by bioinformatic techniques. In all cases, these variants were found in heterozygous form, and most were located in functionally important domains of the protein: the Ca^++^ binding domain, which is crucial for the main function of the ALP protein (p.Arg223Gln, p.Pro307Leu); the crown domain, involved in binding to collagen (p.Arg391Cys, p.Arg450Cys); and the homodimeric domain, which binds ALP to form the active homodimer (p.Arg391Cys, p.Arg450Cys). The p.Arg391Cys variant also affects a residue that is thought to be subject to phosphorylation.^22^ The two novel variants identified in our study, p.Tyr101X and p.Arg301Trp, were also classified as pathogenic based on the criteria suggested by the American College of Medical Genetics and the Association for Molecular Pathology.^23^ In another 4 cases, a missense variant was observed (p.Arg152His). This change has been previously reported as a polymorphism in HPP cases but associated with low ALP and low BMD in a previous study.^12^ It also affects the residue 152, a mutation hotspot also harboring p.Arg152Cys, which has previously been reported to worsen an odonto‐HPP phenotype.^18^ In our study, the p.Arg152His variant was overrepresented in people with low ALP in both cohorts and was associated with a reduced ALP concentration in the LBC1936 cohort. Because many individuals who carried the p.Arg152His polymorphism had ALP values in the normal range, it seems likely that in a subgroup of individuals, other environmental or genetic regulators of ALP may have acted in combination with this polymorphism to push ALP values below the lower limit of normal. Whatever the underlying mechanism, the data presented here support the view that p.Arg152His may be a functional variant that impairs ALP activity. Altogether, our results are consistent with previous findings of adults with persistently low levels of ALP, where 50% to 84% of individuals were found to carry coding variants in *ALPL*.^24^, ^25^

Some missense variants in *ALPL* have been proposed to exert dominant negative effect on enzyme function, especially those located within functional domains.^9^ However, only the p.R391C variant has been tested for this in vitro with negative results.^19^ The results reported here support the view that the missense variants impair ALP function, but further research will be required with each variant to determine if this is due to a dominant negative effect or haploinsufficiency.

Within the clinic population, patients with low ALP tended to have lower BMD values than clinic controls with normal ALP. Although the differences were not significant, the number of patients with low ALP was small. Those with low ALP also had slightly lower serum albumin concentrations but did not have any other distinguishing clinical or biochemical characteristics. It is of interest, however, that missense variants in *ALPL* were significantly overrepresented in the osteoporosis clinic population with low ALP values compared with the general population, indicating that these variants may be a predisposing factor for osteoporosis.

To date, the only treatment approved for patients with HPP is bone‐targeted enzyme‐replacement therapy with asfotase alfa.^9^ Bisphosphonates are generally considered to be contraindicated in HPP because they can inhibit mineralization and because bisphosphonate treatment of adult patients with mild HPP has been associated with atypical femoral fractures.^6^, ^9^, ^26^, ^27^ In this study, 10 patients with potentially damaging *ALPL* variants and osteoporosis had been treated with bisphosphonates and were followed up for an average of just over 6 years. Although the sample size was small, we found no evidence to suggest that patients with *ALPL* variants treated with bisphosphonates fared any worse than those without these variants, given that the number of low‐trauma fractures during follow‐up were similar in the two groups. One patient developed an atypical femoral fracture while on bisphosphonate therapy, but that individual had a normal level of ALP and had no deleterious variants in *ALPL*. Nonetheless, we think it would be appropriate to be cautious in treating patients who have low ALP with using bisphosphonates until further information is available on clinical outcomes from larger cohorts of patients to confirm whether this is the optimal mode of treatment for these patients.

## Disclosures

During the conduct of the study, PR holds a grant from Age UK; SEH holds a grant from the Medical Research Council; IJD holds grants from Age UK, the Medical Research Council, and the Biotechnology and Biological Sciences Research Council; and SHR holds a grant from the European Commission. SHR has acted as an investigator in research studies supported by Amgen, Eli Lilly, Novartis, and Pfizer unrelated to the submitted work. All other authors state that they have no conflicts of interest.

## Supporting information


**Supplemental Table S1.** Primer Sequences for *ALPL* Mutation Screening
**Supplemental Table S2.** In Silico Analysis of the p.Arg301Trp Mutation
**Supplemental Table S3.** Pathogenicity of *ALPL* Variants Found in Osteoporotic Patients With Low ALP Levels
**Supplemental Fig. S1.** Protein sequence alignment of *ALPL* across species.
