# Supplementary material for: Loss‐of‐Function Mutations in the ALPL Gene Presenting with Adult Onset Osteoporosis and Low Serum Concentrations of Total Alkaline Phosphatase
Source: J Bone Miner Res. 2020 Jan 7;35(4):657–61. doi: 10.1002/jbmr.3928 (PMC9328664; doi:10.1002/jbmr.3928)
Supplement: Supplementary file 1 — Supplemental Table S1. Primer Sequences for ALPL Mutation Screening Supplemental Table S2. In Silico Analysis of the p.Arg301Trp Mutation Supplemental Table S3. Pathogenicity of ALPL Variants Found in Osteoporotic Patients With Low ALP Levels Supplemental Fig. S1. Protein sequence alignment of ALPL across species. [file JBMR-35-657-s001.docx]

**Supplementary Information**

**Loss-of-function mutations in the *ALPL* gene presenting with adult onset osteoporosis and low serum concentrations of total alkaline phosphatase**

Nerea Alonso ^1^, Beatriz Larraz-Prieto ^1^, Kathryn Berg ^1^, Zoe Lambert ^1^, Paul Redmond ^2^, Sarah E Harris ^2,3^, Ian J Deary ^2,3^, Carys Pugh ^4^, James Prendergast ^5^, Stuart H Ralston ^1^

^1^ Rheumatology and Bone Disease Unit, CGEM-IGMM, University of Edinburgh, Edinburgh, UK

^2^ Department of Psychology, University of Edinburgh, 7 George Square, Edinburgh, UK

^3^ Centre for Cognitive Ageing and Cognitive Epidemiology, University of Edinburgh, Edinburgh, UK

^4^ Division of Psychiatry, University of Edinburgh, Royal Edinburgh Hospital, Edinburgh, UK

^5^ The Roslin Institute, University of Edinburgh, Easter Bush, Midlothian, UK

**Methods**

**Mutation Screening of *ALPL***

Mutation screening of *ALPL* (RefSeq NM_000478) in clinic cases was conducted on genomic DNA extracted from venous blood using the QIAAmp DNA blood extraction kit (Qiagen, GmbH, Hilden, Germany). All 11 coding exons of *ALPL* and the intron-exon boundaries were amplified by PCR with primers pairs designed using Primer3 v4.0.0 software ^(1,2)^. The primer sequences are shown in Table S1. The PCR products were screened for variants using Sanger sequencing according to standard techniques. All variants found were confirmed by sequencing of two independent PCR products. Mutation screening in the LBC1936 cohort was performed by whole genome sequencing. Genotypes for p.Val522Ala (rs34605986) variant were extracted from the Illumina 610 quad array 1000 genomes imputed dataset. The variant was imputed with an imputation quality score of 1.0. Genotypes for p.Arg152His variant (rs149344982) were extracted from the Illumina HiSeq X whole genome sequencing data set which provided 30x depth of coverage.

***In silico* analysis of *ALPL* mutations**

Pathogenicity of the novel p.Arg301Trp missense variant was assessed using a variety of freely-available software tools: PolyPhen-2 ^(3)^, PROVEAN ^(4)^, SIFT ^(5)^, Pmut ^(6)^, PANTHER ^(7)^, Align-GVGD ^(8)^, MutationTaster ^(9)^, SNPS3D ^(10)^, and PSIPRED ^(11)^. Clustal Omega software ^(12,13)^ was used to check the conservation status of the region where the missense substitution was found. The results of this analysis were consistent with a pathogenic mutation as summarised in Suppl. Table S2. For the remaining mutations pathogenicity was assessed using the criteria proposed by the American College of Medical Genetics and Genomics and the Association for Molecular Pathology ^(3)^. These results are summarised in Suppl. Table S3.

**Suppl. Table S1. Primer sequences for *ALPL* mutation screening**

| **Exon** | **Forward** | **Reverse** | **Product size (bp)** |
| --- | --- | --- | --- |
| 2 | ctgtaataggtgctcaccga | agacactgccctcatcatac | 350 |
| 3 | ctgtacgtctggagatagga | ttcaacacagcccccttcaa | 274 |
| 4 | actagagagcttctgggtac | tctggctgctgtcatgttca | 277 |
| 5 | agtccccatggtgtgagtgta | agaaagactgaggcctggaca | 324 |
| 6 | tacttggaagccactgccaga | aaccgcaaatcccctaatggg | 501 |
| 7 | gaaagtgtccacaccatctc | ggacaacagaacttcagagc | 344 |
| 8 | gtaaaggcctcagactctga | gcctaattccaggaaccaga | 334 |
| 9 | agcttccttggagtcctccta | agagctggacttctccatcct | 280 |
| 10 | aatccagcagcagtgttgtg | ttgtcattgagtccccacca | 389 |
| 11 | aagaagatcccaggggttac | tgaccttgaccacaagctga | 316 |
| 12 | agggagatggaaaagctgcgt | tgtgggaagttggcatctgtc | 393 |

**Suppl. Table S2.** ***In silico* analysis of the p.Arg301Trp mutation.**

| **Software** | **Score** | **Prediction** |
| --- | --- | --- |
| PolyPhen-2 | 1 | Probably damaging |
| SIFT | 0 (median 2.69) | Damaging |
| PROVEAN | -7.43 | Deleterious |
| Pmut | 0.726 (reliability 4) | Pathological |
| PANTHER | -3.29321 (P 0.57278) | Deleterious |
| Align-GVGD | GV = 0.00 / GD = 101.29 | Pathological |
| MutationTaster | N/A | Disease causing |
| SNPS3D | -2 | Pathogenic |
| PSIPRED | N/A | Coil structure |

**Suppl. Figure S1. Protein sequence alignment for *ALPL* across species**

Amino Acid: 290 300 310 320

| | | |

Danio rerio: lfepadlnyelerntendpsltemvdvaikil

Xenopus tropicalis: lfepidmlyelernttmdpslpemvemaikil

Gallus gallus: lfepgdmvyeldrnnetdpslsemvavairml

Mus musculus: lfepgdmqyelnrnnltdpslsemvevalril

Rattus norvergicus: lfepgdmqyelnrnnltdpslsemvevalril

Macaca mulatta: lfepgdmeyelnrnnvtdpslsemvvvaiqil

Human: LFEPGDMQYELNRNNVTDPSLSEMVVVAIQIL

Pan paniscus: lfepgdmqyelnrnnvtdpslsemvvvaiqil

Pan troglodytes: lfepgdmqyelnrnnvtdpslsemvvvaiqil

Eqqus caballus: lfepgdmqyelnrnnvtdpslsemvemaikil

Bos taurus: lfepgdmqyelnrnnatdpslsemvemairil

Sus scrofa: lfepgdmqyelnrnnvtdpslsemvemairil

Felis catus: lfepgdmqyelnrnsttdpslsemveiaikil

Vulpes vulpes: lfepgdmqyelnrnnvtdpslsemmeiaikil

Canis lupus familiaris: lfepgdmqyelnrnnvtdpslsemveiaikil

Conserved: **** *: ***:**. **** **: :*:::*

The protein sequence alignment across species between amino acids 289 and 320 of the alkaline phosphatase protein is shown. The site of the p.Arg301Trp mutation is highlighted by red text. Strictly conserved positions are indicated as * and partially conserved positions are indicated as . (conservation between groups of weakly similar properties; < 0.5 in the Gonnet PAM 250 Matrix) or : (conservation between groups of strongly similar properties; > 0.5 in the Gonnet PAM 250 Matrix). The following species were tested: zebrafish (Danio rerio), Western clawed frog (Xenopus tropicalis), red junglefowl (Gallus gallus), house mouse (Mus musculus), brown rat (Rattus norvergicus), Rhesus macaque (Macaca mulatta), human, bonobo (Pan paniscus), chimpanzee (Pan troglodytes), horse (Eqqus caballus), cow (Bos taurus), wild boar (Sus scrofa), cat (felis catus), red fox (Vulpes vulpes), and domestic dog (Canis lupus familiaris).

**Table S3. Pathogenicity of *ALPL* variants found in osteoporotic patients with low ALP levels.**

|  | **Criteria for classifying pathogenic variants** | | | |  |
| --- | --- | --- | --- | --- | --- |
| ***ALPL* variant** | **Very strong evidence** | **Strong evidence** | **Moderate evidence** | **Supporting evidence** | **Classification** |
| p.Tyr101X | PVS1 | PS1, PS4 | PM2 | - | Pathogenic |
| p.Arg152His | - | PS1, PS4 | PM2 | - | Pathogenic |
| p.Glu146Lys | - | PS1, PS3, PS4 | PM2 | PP5 | Pathogenic |
| p.Met192Thr | - | PS1, PS4 | PM2 | PP5 | Pathogenic |
| p.Arg223Gln | - | PS1, PS4 | PM1, PM2 | PP5 | Pathogenic |
| p.Pro307Leu | - | PS1, PS4 | PM1, PM2 | PP5 | Pathogenic |
| p.Arg301Trp | - | PS4 | PM1, PM2 | PP3 | Pathogenic |
| p.Val374Met | - | PS1, PS4 | PM2 | PP5 | Pathogenic |
| p.Arg391Cys | - | PS1, PS4 | PM1, PM2 | PP5 | Pathogenic |
| p.Arg450Cys | - | PS1, PS4 | PM1, PM2 | PP5 | Pathogenic |

Pathogenicity was assessed using the criteria adopted by a joint consensus recommendation of the American College of Medical Genetics and Genomics and the Association for Molecular Pathology ^(14)^. PVS1: null variant in a gene where loss of function is a known mechanism of disease; PS1: Same amino acid change as a previously established pathogenic variant regardless of nucleotide change; PS3: Well-established *in vitro* or *in vivo* functional studies supportive of a damaging effect on the gene or gene product; PS4: prevalence of the variant in affected individuals is significantly increased compared to the prevalence in controls; PM1: located in a mutational hot spot and/or critical and well-established functional domain without benign variation; PM2: absent from controls (or at extremely low frequency if recessive) in Exome Sequencing Project, 1000 Genomes or ExAC; PP5: Reputable source recently reports variants as pathogenic but the evidence is not available to the laboratory to perform an independent evaluation.

**References**

1. Koressaar T, Remm M. Enhancements and modifications of primer design program Primer3. Bioinformatics. 2007;23(10):1289-91.

2. Untergasser A, Cutcutache I, Koressaar T, et al. Primer3--new capabilities and interfaces. Nucleic Acids Res. 2012;40(15):e115.

3. Adzhubei IA, Schmidt S, Peshkin L, et al. A method and server for predicting damaging missense mutations. Nat Methods. 2010;7(4):248-9.

4. Choi Y, Chan AP. PROVEAN web server: a tool to predict the functional effect of amino acid substitutions and indels. Bioinformatics. 2015;31(16):2745-7.

5. Kumar P, Henikoff S, Ng PC. Predicting the effects of coding non-synonymous variants on protein function using the SIFT algorithm. Nat Protoc. 2009;4(7):1073-81.

6. Ferrer-Costa C, Gelpi JL, Zamakola L, et al. PMUT: a web-based tool for the annotation of pathological mutations on proteins. Bioinformatics. 2005;21(14):3176-8.

7. Mi H, Muruganujan A, Thomas PD. PANTHER in 2013: modeling the evolution of gene function, and other gene attributes, in the context of phylogenetic trees. Nucleic Acids Res. 2013;41(Database issue):D377-86.

8. Tavtigian SV, Deffenbaugh AM, Yin L, et al. Comprehensive statistical study of 452 BRCA1 missense substitutions with classification of eight recurrent substitutions as neutral. J Med Genet. 2006;43(4):295-305.

9. Schwarz JM, Rodelsperger C, Schuelke M, Seelow D. MutationTaster evaluates disease-causing potential of sequence alterations. Nat Methods. 2010;7(8):575-6.

10. Yue P, Melamud E, Moult J. SNPs3D: candidate gene and SNP selection for association studies. BMC Bioinformatics. 2006;7:166.

11. Jones DT. Protein secondary structure prediction based on position-specific scoring matrices. J Mol Biol. 1999;292(2):195-202.

12. Goujon M, McWilliam H, Li W, et al. A new bioinformatics analysis tools framework at EMBL-EBI. Nucleic Acids Res. 2010;38(Web Server issue):W695-9.

13. Sievers F, Wilm A, Dineen D, et al. Fast, scalable generation of high-quality protein multiple sequence alignments using Clustal Omega. Mol Syst Biol. 2011;7:539.

14. Richards S, Aziz N, Bale S, et al. Standards and guidelines for the interpretation of sequence variants: a joint consensus recommendation of the American College of Medical Genetics and Genomics and the Association for Molecular Pathology. Genet Med. 2015;17(5):405-24.
